# Sex-dependent Lupus *Blautia (Ruminococcus) gnavus* strain induction of zonulin-mediated intestinal permeability and autoimmunity

**DOI:** 10.3389/fimmu.2022.897971

**Published:** 2022-08-11

**Authors:** Gregg J. Silverman, Jing Deng, Doua F. Azzouz

**Affiliations:** Laboratory of B Cell Immunobiology, Department of Medicine, NYU Grossman School of Medicine, New York, NY, United States

**Keywords:** autoimmunity, microbiome, lupus, lipoglycan, FMT, *Ruminococcus gnavus*, leaky gut, pathobiont

## Abstract

Imbalances in the gut microbiome are suspected contributors to the pathogenesis of Systemic Lupus Erythematosus, and our studies and others have documented that patients with active Lupus nephritis have expansions of the obligate anaerobe, *Blautia (Ruminococcus) gnavus* (RG). To investigate whether the RG strains in Lupus patients have *in vivo* pathogenic properties in a gnotobiotic system, we colonized C57BL/6 mice with individual RG strains from healthy adults or those from Lupus patients. These strains were similar in their capacity for murine intestinal colonization of antibiotic-preconditioned specific-pathogen-free, as well as of germ-free adults and of their neonatally colonized litters. Lupus-derived RG strains induced high levels of intestinal permeability that was significantly greater in female than male mice, whereas the RG species-type strain (ATCC29149/VPI C7-1) from a healthy donor had little or no effects. These Lupus RG strain-induced functional alterations were associated with RG translocation to mesenteric lymph nodes, and raised serum levels of zonulin, a regulator of tight junction formation between cells that form the gut barrier. Notably, the level of Lupus RG-induced intestinal permeability was significantly correlated with serum IgG anti RG cell-wall lipoglycan antibodies, and with anti-native DNA autoantibodies that are a biomarker for SLE. Strikingly, gut permeability was completely reversed by oral treatment with larazotide acetate, an octapeptide that is a specific molecular antagonist of zonulin. Taken together, these studies document a pathway by which RG strains from Lupus patients contribute to a leaky gut and features of autoimmunity implicated in the pathogenesis of flares of clinical Lupus disease.

## Introduction

Our gut microbiomes contain complex interdependent communities that in health provide layered tiers for nutritional and immune regulatory benefits. Imbalances (or dysbiosis) have been implicated in a growing number of clinical conditions but only in a handful of cases have expansions of specific bacteria been implicated, and in even fewer cases have actual pathogenic pathways been identified. In studies of a clinically diverse cohort of female patients with Systemic Lupus Erythematosus (SLE), we discovered the first correlation between overrepresentation of an obligate anaerobe, *Blautia* (*Ruminococcus) gnavus* (RG) with SLE disease activity (1). More intriguingly, even amongst Lupus patients these RG expansions were more common in patients with Lupus nephritis, which affects more than half of patients and is associated with great morbidity and mortality (1). Notably, the association of RG with LN was also later independently documented in a large cohort of untreated Chinese Lupus patients (2). In more recent longitudinal studies of our cohort, Lupus patients were found to have inherently unstable gut microbiota communities and almost half of clinical flares of renal disease were temporally associated with ephemeral RG blooms (manuscript submitted).

One of the estimated 53 most common human intestinal colonizers by metagenomic sequencing (3), *RG* are early colonizers that are detectable in most infants by 24 months of age (4). In adults, RG is present in at least 90% of individuals from North American and Europe, although generally at stable low-levels at or below 0.1% abundance (3, 5).

Based on genomic phylogenetic analysis, RG has been reassigned to the Phylum Firmicutes, family Lachnospiraceae and genus Blautia of spore-forming obligate anaerobes. As a species, RG is quite distinct from other taxa at both the genome as well as for 16S rRNA gene sequence level (6). In healthy adults, RG plays pleiotropic roles in host metabolism and immunity [reviewed in (7)], including for the conversion of primary to secondary bile acids (8), production of the short-chain fatty acids (SCFAs) that aid immune regulation (9), and hence most strains are considered pro-homeostatic.

In general, most reports of disease-associated RG abundance variations in microbiota communities have been limited to correlative studies, with limited exception (10, 11). The *in vivo* effects of RG isolates on host immunity have largely focused on the Human Microbiome Project designated type-specific RG strain, VPI C7-9, also termed ATCC29149 (referred to in our studies as RG1) (11–13) isolated from stool of a healthy donor (14). While increased intestinal abundance of the RG species has been associated with Lupus disease flares (1), we hypothesized that there may be important differences in the pathogenetic potential of strains from healthy individuals from those colonizing LN patients. Moreover, we have discovered that active Lupus Nephritis patients have gut RG expansions (1), with concurrent high serum IgG antibodies to a novel lipoglycan which we have discovered is produced by RG strains (termed S107-48 and S47-18) that provide circumstantial evidence of involvement in autoimmune pathogenesis.

We therefore set out to compare the effects of different genome-defined RG strains following *in vivo* intestinal colonization, with an emphasis on assessing whether gut-barrier function was affected. Whereas we found that all of the RG strains had the capacity to colonize the mouse gut, there were dramatic differences in the effects of individual strains on intestinal permeability, which was found to be mediated by a zonulin-dependent mechanism. Indeed, strains isolated from clinically active Lupus patients reproducibly induced these changes. Moreover, neonatal murine colonization with a Lupus RG strain resulted in microbial translocation and systemic antibody responses to RG-specific antigens and induction of Lupus autoantibodies. These findings provide a mechanistic rationale for the previously reported linkage between RG intestinal expansions and Lupus pathogenesis (1).

## Materials and methods

### Generation of an RG-specific lipoglycan specific murine monoclonal antibody

Fecal samples from two female lupus nephritis patients (termed S47 and S107), were obtained at time of disease flare, documented by high-level urine protein creatinine ratios and high scores on the SLEDAI disease activity index (15) of 8 and 22, respectively. By 16S rRNA amplimer analysis (1), the fecal samples of healthy controls had a mean RG level of 0.1%, whereas the S47 and S107 lupus nephritis patients had increased RG abundance of 2.3% and 3.1%, respectively. These fecal samples were streaked onto bacterial media plates. Based on morphology and growth characteristics, individual colonies were selected, and screened by 16S rRNA specific PCR amplification using oligos with genomic DNA sequences able to the *Blautia (Ruminococcus) gnavus* (RG) species, (16), which was confirmed by whole genome sequence determination and analysis. By this approach the Lupus-derived RG strains, S47-18 and S107-48 were identified. Raw sequence data and assemblies have been deposited in NCBI under BioProject PRJNA82122).

**Figure 1:**
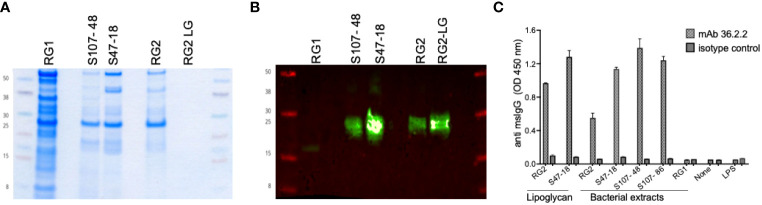
Immunoreactivity and binding specificity of the murine monoclonal antibody 36.2.2 to the lupus RG strains associated lipoglycan. **(A)** Coomassie stain for one of duplicate gels loaded equally with the whole extracts of a reference strain (RG1) that lacks the lipoglycan (LG, the two lupus RG strains (S107-48, S47-18) expressing LG, the reference strain (RG2) and LG purified from this strain (RG2-LG). **(B)** Immunoblot with the mAb 36.2.2 using the other duplicate gel as in **(A)**, the blot is showing the reactivity only to the LG band in the two RG lupus strains (S107- 48, S47-18) as well as the reference strain (RG2) and its purified LG. **(C)** Immunoreactivity by ELISA reflected as ODs of the mAb 36.2.2 binding to all extracts and to both purified LGs from RG2 and the lupus strain S47-18 but not the LPS from *Pseudomonas*.

To generate RG-specific monoclonal antibodies, 10 BALB/c mice were immunized by a vendor (Envigo) with an extract of the (the BEI strain CC55_001C), which we have termed the RG2 strain, emulsified in complete Freund’s adjuvant and then 14-days later boosted with lipoglycan (LG) purified from the Lupus S47-18 strain emulsified in incomplete Freund’s adjuvant, This strategy has been used in phage display selection of antibody clones with antigenic cross-reactivity of interest (17). The cells from the mouse with the strongest post-immunization response were fused with NS-1 myeloma cells. After subcloning, the spent supernatants after 7 days were evaluated for IgG-reactivity, which demonstrated highly correlated reactivity with whole extracts of the immunizing RG strain and then with RG lipoglycan, by the subcloned hybridoma cell line, termed mAb 36.2.2. By immunoblot, this mAb was reactive with oligo-bands that migrate in the same range as 20-30 kilodalton protein markers, and these bands also display equivalent Lupus serum IgG-reactivity with oligo-bands in the lipoglycan producing RG strains, RG2, S107-48 and S47-18 (1) while these bands are absent in the RG1 strain (**Figure 1**). Specificity was also confirmed by ELISA, when the plates were coated with bacterial extracts from RG2, S47-18, S107-48, S107-86, RG1, the purified lipoglycan from RG2 and S47-18 as well as with LPS from *Pseudomonas aeruginosa* as a control from other species. Next, the murine monoclonal 36.2.2 was added at 2 concentrations (at 100ng/ml and 25ng/ml) in duplicate each incubated for 2hs at RT. Binding was detected by goat anti mouse IgG HRP conjugate at a 1:10,000 dilution (Jackson ImmunoResearch™), the TMB substrate was added to develop the plate.

### Mice

All murine colonization work was performed under the supervision of the NYU Langone IACUC. Germ-free (GF) mice were bred and maintained in the gnotobiotic facility at the NYU Langone (18). All mice were C57BL/6 genotype, and locally bred or purchased from Charles River Laboratories (Wilmington MA) and were received at 6–8 weeks of age, or locally bred. All other mice were, maintained in specific-pathogen-free (SPF) cages, with free access to food and water. To avoid possible cross-contamination mice colonized with individual strains were separately raised in isolator cages. Mice were housed under a 12hr light/dark cycle at 23°C.

### Intestinal colonization with individual RG strains

Individual *RG* strains were streaked then individual colonies grown in 5 ml of BHI media (Anaerobe Systems) under anaerobic conditions overnight. GF C57BL/6 mice (4 male and 4 female) were colonized by oral gavage with different *RG* strains (RG1, RG2 or S107-48) of 10^8^ CFU in 500 μL of sterile PBS, every other day for a total of five times. Individual mice were weighed, then fecal pellets and bleeds were collected from individual mice prior to gavage, and again at days 7, 14, and 21 following gavage. Pellets were stored at −80°C until DNA extraction. Bacterial translocation and burden of *R. gnavus* in feces was, determined by RG-specific qPCR at the indicated time points. A male and a female colonized with the same RG strain were then bred, and from litters fecal pellets obtained from individual pups, after weaning , for evaluation of RG colonization. Birth, litters were cohoused with their individual dams to ensure sharing the RG strains colonization from the fecal pellets from their mother.

To colonize mice previously raised under Specific Pathogen-Free (SPF) conditions, 4-6 week old mice were preconditioned with oral antibiotics. The antibiotic cocktail was composed of vancomycin (0.5g/L)(Fisher Scientific), neomycin (1g/L)(Fisher Scientific), ampicillin (1g/L; Fisher Scientific) and metronidazole (1g/L; Fisher Scientific), with solutions freshly prepared each week in autoclaved drinking water, and all antibiotics remained soluble at this concentration. Antibiotics were provided in 100-ml clear glass sippers (Braintree Scientific, Inc., Braintree MA). SPF mice received systemic antibiotics (see above) at 4 weeks of age for one month. Fecal pellets were collected prior to, and following antibiotic exposure at days 21, and then weekly until at least 100-fold decrease of total 16S rRNA (representing bacterial burden) in fecal pellets by qPCR was confirmed for each mouse. Mice were then switched to plain water *ad libitum* for 24 hours, then mice received as above described, oral gavage with different *RG* strains (RG1, RG2, S107-48 or S47-18) (see **Supplementary Figure 1**). A male and female colonized with the same strain were bred, and after weaning of litters fecal pellets were collected to evaluate colonization. All strains, except RG1, yielded litters.

### Quantitative PCR analysis

For bacterial genomic 16S rRNA gene quantitation, DNA was isolated from mice fecal pellets, and from cecum contents, using QIAamp DNeasy Powersoil spleen and mesenteric lymph nodes we used the kit. Blood & Tissue kit (Qiagen), according to the manufacturer’s instructions. Also, from spleens and mesenteric lymph nodes (MLNs) using QIAamp Blood & Tissue DNA Mini Kit. Briefly, small tissue pieces were placed in 1.5ml microcentrifuge tubes followed by adding 180μl of buffer ATL and 20μl Proteinase K, mixed by vortexing and incubated at 56°C until completely lysed (1–3 h). The additional steps to purify the DNA were performed according to the kit instructions.

DNA isolated from all these sample was quantified on a Nanodrop 1000 (Thermo Scientific) and then run for quantitative PCR assays on the StepOnePlus™ Real-Time PCR System (Applied Biosystems) was performed with the Power SYBR Green Master Mix (Applied Biosystems). For end-point PCR reactions, a thermal cycler (Applied Biosystems) was programmed to amplify bacterial DNA using the following primers that were also used for qPCR experiments. PCR reactions were run with the following conditions: 95°C for 5 minutes followed by 50 cycles of 95°C for 15 seconds, 58°C for 30 seconds; and followed by extension at 72°C for 5 minutes. Total bacterial 16S rRNA gene content was assessed with the oligonucleotide primers (16):

UniF340(5’-ACTCCTACGGGAGGCAGCAGT-3’)
UniR514(5’-ATTACCGCGGCTGCTGGC-3’).

The RG species-specific 16S rRNA was determined with previously reported oligonucleotide primers (16):

Fwd 5’-GGACTGCATTTGGAACTGTCAG-3’
Rev 5’-AACGTCAGTCATCGTCCAGAAAG-3’

### *In vivo* assay of intestinal permeability

To assess intestinal permeability, after 4 h fasting, mice were orally gavaged with 4,000-Da fluorescein (FITC)-dextran (FD4) (Sigma-Aldrich, St. Louis, MO) (250 mg/kg body weight) in 200 μl buffered saline, and blood was then collected 3 hr later. The concentration of the FD4 was determined using a fluorimeter with an excitation wavelength at 490 nm and an emission wavelength of 530 nm. To assess concentration, FD4 in serum was then serially diluted to establish a standard curve.

### Histopathology studies

Intestines were emptied of contents and formalin fixed for further examination using unstained and H&E staining. Sterilely isolated portions of liver and ileal biopsies were streaked on Chopped Meat Media and incubated in an anerobic chamber but no colonies with morphology of *R. gnavus* were detected after 14 days.

### Leaky gut normalization studies

Zonulin antagonist, larazotide acetate (also known as AT-1001, or INN-202), was purchased from BOC Sciences, NY). Individual neonatally-colonized littermates from SPF mice, colonized with different RG strains, were retested after FD4 challenge, and those with abnormal levels each then received 0.15 mg/ml of the zonulin antagonist in the drinking water, which was refreshed every day, for 10 consecutive days. After a 24 hr rest, intestinal permeability was then retested.

### Assays of antigen-reactive IgG antibodies

A custom multiplex bead-based array using the Magpix platform (Luminex, Austin TX) was created by coupling a variety of highly purified ligands, including thymic native DNA, purified cell wall lipoglycan (LG) from the RG2 strain [formerly termed LG3 (1)], and the Lupus RG strain S47-18 LG, recombinant *S. aureus* proteins, endotoxins, and other bacterial antigens and control ligands, to individual microspheres, adapting the manufacturer’s protocol and previous reports (19–21). For antigen-reactive IgG detection, 1,000 microspheres per analyte per well were premixed, sonicated, and then with addition of 100 μl of diluted serum was added, as indicated. For ELISA bound IgG, antibodies were detected with Fc-gamma-specific anti-murine IgG HRP (eBioscience, San Diego CA), and for multiplex bead-based assay detection with anti-mouse IgG (Fc-specific) *F*(ab′)2 PE (Jackson ImmunoResearch, West Grove PA). Data were acquired on a Magpix instrument (Luminex) and reported as mean fluorescence intensity (MFI) values, as previously described (19–21). Plasma zonulin levels were quantitated with a commercial ELISA Kit. (MyBioSource, San Diego CA.)

### Biostatistics

Comparisons were with two-tailed unpaired or paired t-tests, or Spearman correlations, as indicated, with Prism version 9.0 for Mac OS (Graphpad, San Diego CA). p<0.05 was significant.

## Results

### RG strains are subgrouped based on expression vs. non-expression of a conserved lipoglycan

In these studies, we sought to investigate for differences in the *in vivo* pathogenicity properties of *Blautia (Ruminococcus) gnavus* (RG) strains isolated from a fecal sample of a healthy donor, VPI C7-9 (here termed RG1) and from a colonic biopsy, CC55_001C (here termed RG2), with comparisons to the RG strains, S107-48 and S47-18 obtained from two SLE patients with RG blooms at the time of flare of renal disease.

To provide an independent approach to investigate the antigenic diversity expressed by different RG strains, we generated murine monoclonal antibodies by RG bacterial immunization (see *Methods*). By ELISA, the resultant mAb 36.2.2 was strongly reactive with the purified LGs from the S47-18 strain used for the immunization boost as well as the previously reported purified LG from the RG2 strain (**Figure 1**). In addition, this mAb was reactive with whole extracts of the RG2 strain and Lupus strains, S47-18, S107-48 and S107-86, that were isolated from the two different LN patients (**Figure 1**). This antibody, however, was non-reactive with the RG1 type strain. RG strains were therefore assignable to two subgroups, based on the presence or absence of a cell wall associated lipoglycan with the same MW oligoband distribution and shared antigenic determinants that are recognized by the mAb 36.2.2. Furthermore, the binding specificity of this antibody was confirmed by ELISA reflected by high ODs found for all strains expressing LG and to the purified LG, while no binding was detected to RG1 and to LPS from another bacterial species *Pseudomonas aeruginosa*.

### RG persistence in germ-free mice with transmission to litters

**Figure 2:**
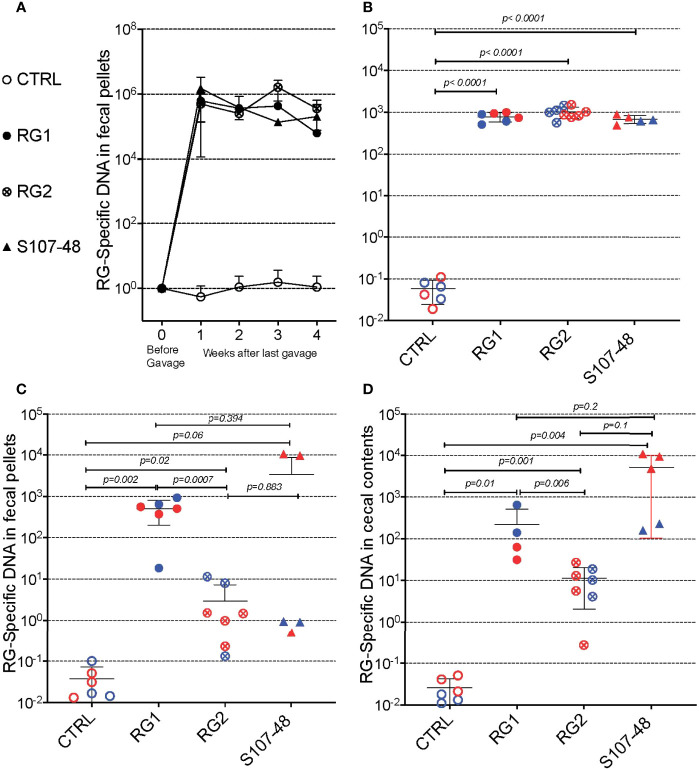
Diverse RG strains isolated from human donors colonize germ-free (GF) mice, and are passed to their litters. **(A)** Abundance of RG DNA in germ-free (GF) C57BL/6 mice breeding pairs, monocolonized by oral gavage with RG1, RG2 or S107-48 RG strains. RG species-specific 16S rRNA genomic levels were quantified by genomic qPCR analysis of fecal pellets, as shown. Inset values indicate normalized quantity of RG 16S rDNA with control mice, before (left) and weeks after (right) gavage with each of the RG stains, as indicated. **(B)** Abundance of RG-specific genomic DNA in 4wk-old litters of the RG1, RG2 and S107-48-monocolonized GF breeding pairs. RG species-specific 16S rRNA levels were quantified by genomic qPCR analysis of fecal pellets, as shown. **(C)** Abundance of RG DNA in fecal pellets of 12-14wks-old litters of the individual RG1, RG2 and S107-48 strains monocolonized GF breeding pairs. **(D)** Abundance of RG DNA in cecal pellets of 12-14 wk-old litters of RG1, RG2 and S107-48-monocolonized GF breeding pairs. Note that overtime there is increased variability of RG strain persistence as measured in fecal pellets, but less heterogeneity in RG representation in the luminal extracts of the cecums of these same individual colonized mice. (Red) indicates female and (Blue) male mice are indicated separately.

To initiate these studies, in a gnotobiotic system we used a standard gavage protocol to colonize groups of germ-free C57BL/6 mice with different RG strains. Due in part to the practical challenges of accurate CFU determinations from this obligate anaerobe we adapted a previous described RG rRNA species genomic-specific qPCR assay that was validated to be highly RG species specific (16). Whereas in the fecal pellets of naïve germ-free C57BL/6 mice RG was undetectable, following gavage with different RG strains, high levels were detectable in sequential samples obtained at four weeks following gavage (**Figure 2A**). There initially were no differences in levels of fecal RG representation with the different strains, nor differences based on the sex of the recipient mice (**Figures 1A**, **2A**, and data not shown).

Earlier studies in mice have demonstrated that adult germ-free mice have immune defects, whereas colonization of neonatal mice results in exposure to antigens of colonizing bacterial species that can affect both host innate immunity, as well as lymphocyte development (22). We therefore mated breeding pairs of these formerly GF mice that had each been colonized with individual RG strains. Each of these strain-colonized pairs yielded litters, which were then weaned. Individual fecal samples were collected and testing showed roughly equivalent high levels of RG-species specific 16S rRNA genes (**Figure 2B**).

To investigate for the persistence of RG colonization, fecal pellets were also obtained at about 3-months of age, and RG levels were found to diverge based on the colonizing RG strains. In specific, compared to controls highest mean levels were found in mice colonized with the RG1 strain (*p*=0.002), with lower but still significant levels were detected in mice colonized with the RG2 strain (*p*=0.02) (**Figure 2C**). Strikingly, although each was above the level found in the non-colonized control mice, the RG levels detected in S107-48 Lupus strain colonized mice varied greatly (**Figure 2C**), and were quantitatively different compared to controls (p=0.06). Interestingly, in this group fecal RG levels were more heterogeneous, and higher in two of the three females and lower in the two S107-48 colonized male mice (**Figure 2C**).

In health, species of the Lachnospiraceae family are known to differentially colonize regional sites within the intestine (23). Therefore, after sacrifice, the cecal luminal contents were sampled and significant levels of RG-specific genomic DNA were documented in mice colonized with each of the three RG strains (**Figure 2D**). Notably, the highest mean levels were documented for the Lupus S107-48 strain, followed by the RG1 and then the RG2 strain that had been isolated from healthy individuals. Hence, there were differences in the persistence of different strains of this human commensal within individual monocolonized mice.

### RG bacteria efficiently colonizes antibiotic-preconditioned SPF mice

**Figure 3:**
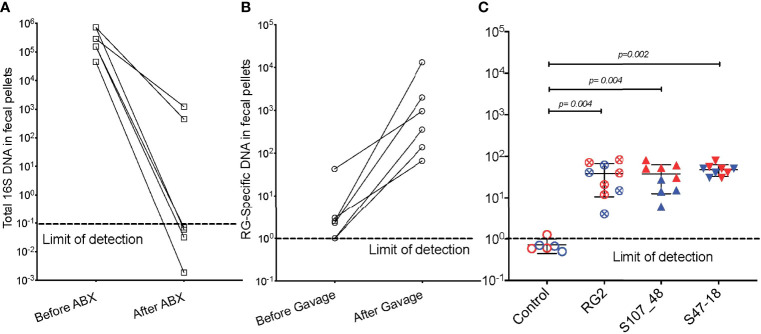
Broad-spectrum oral antibiotic-conditioned mice raised under specific pathogen-free (SPF) conditions were colonized with diverse RG strains isolated from healthy and Lupus-affected human donors. The colonizing RG strains were then passed to their litters. **(A)** The fecal pellets of SPF-raised C57BL/6 breeding pairs were collected before initiating oral regimen of broad-spectrum antibiotics and repeated four weeks later, with total 16S rRNA genomic levels measured by qPCR analysis of extracts of fecal pellets. **(B)** Fecal pellets from these same mice were evaluated for levels of RG-specific genomic DNA, before and after gavage with freshly cultured RG1, RG2 and S107-48 RG strains. **(C)** After mating of pairs based on same RG strain, abundance of RG genomic DNA in fecal pellets of 5wk-old litters was measured in each of the control (sham colonized, 3M 3F), RG2 (4M 5F), S107-48 (4M 4F) and S47-18 (4M and 5F) strain monocolonized SPF littermates, by RG-species specific 16S rDNA genomic DNA by qPCR analysis. (Red) indicates female and (Blue) male mice are separately shown. Comparisons by unpaired *t* test, *p* values shown. Control group was gavaged with sterile PBS.

We also investigated the capacity of these RG strains to colonize immunocompetent adult mice that had been bred and raised under SPF conditions. To precondition the intestinal bacterial burden, we employed a previously validated combination oral antibiotic regimen and to monitor bacterial depletion we utilized a total 16S rRNA qPCR assay proven to amplify bacterial taxa with broad phylogenetic origins (see *Methods*) (**Figure 3A**), By this approach we documented that after a one month treatment duration community abundance was reduced at least 100-fold in the antibiotic-treated mice (**Figure 3A**), and then individual mating pairs were then gavaged with different RG strain cultures. In comparisons of paired fecal pellet samples obtained before and two weeks after gavage, although there was inter-individual variation, the RG-specific qPCR assay demonstrated significant levels of RG colonization in each of the recipient mice (**Figure 3B**). After mating of pairs colonized with the same RG strain, fecal pellets of 5wk-old litters contained abundance of RG genomic DNA in the RG1, RG2, S107-48 and S47-18 strain monocolonized SPF groups were roughly equivalent and significantly greater than in uncolonized control mice in which RG was undetectable (**Figure 3C**). These studies document the capacity of diverse human commensal RG strains to colonize antibiotic-preconditioned mice raised under SPF conditions.

### Lupus RG strains induce increased gut permeability

**Figure 4:**
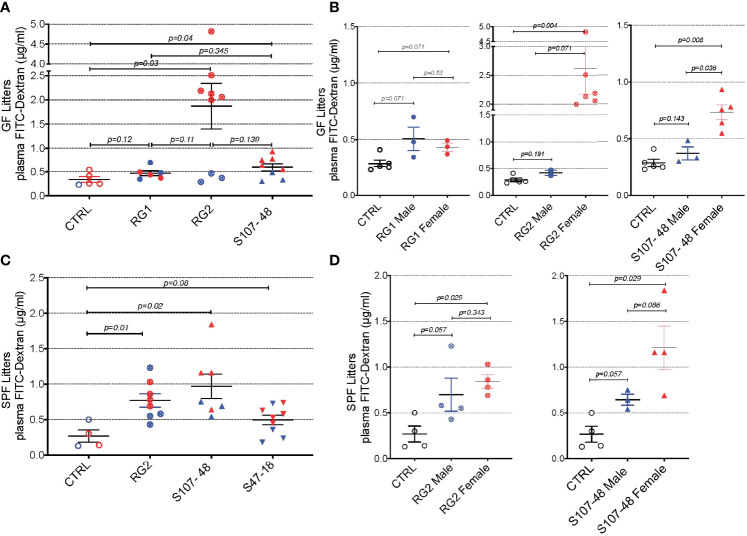
Intestinal colonization of GF and SPF mice by select RG strains induces enhanced intestinal permeability. **(A)** Neonatal intestinal colonization of the litters of GF mice by RG strains, RG2 and S107-48 but not the RG1 strain, induces increased intestinal permeability when tested by the FITC-dextran challenge assay in mice at 10-14 weeks of age. **(B)** Higher levels of intestinal permeability were found in RG2 and S107-48 strain colonized females than male littermates. **(C)** Litters of SPF mice that were colonized by the RG strains, RG2 and S107-48, displayed increased intestinal permeability. Due to heterogeneity in responses, the group colonized by the S47-48 RG strain did not attain significance. **(D)** Female mice colonized with RG2 and S107-48 displayed significantly greater levels of intestinal permeability than male mice or non-colonized control mice. Results indicate mean ± SEM (n= 3- 6 mice per group). In some cases mice were lost to unwitnessed deaths. Comparisons by unpaired *t* test, *p* values shown. Control received sham instillations with sterile PBS.

To investigate the potential influences of RG intestinal colonization on gut barrier integrity, we gavaged individual fasting mice with a standardized dose of FITC-labeled dextran of 4000 Da molecular weight (FD4), then blood samples were subsequently obtained to assess for altered intestinal permeability (**Figure 4**). In the litters from GF mice colonized with RG, little or no leakage was detected in the weaned pups colonized by RG1. In contrast, there was evidence of increased permeability in both the litters colonized with the RG2 strain and the Lupus strain, S107-48 (**Figure 4A**). There were no differences in plasma FD4 levels found in male and female mice colonized with the RG1 strain (**Figure 4B**). Strikingly, the female mice colonized with the RG2 strain and those with the Lupus S107-48 strain had substantially higher levels of post-challenge plasma FD4, consistent with greater intestinal permeability in females colonized with these RG strains (**Figure 4B**).

Mating of the pairs colonized with the different lipoglycan-expressing strains resulted in litters except for the RG1-colonized mice that did not yield progeny. In the litters of RG-colonized SPF mice, we again found evidence of increased gut permeability in the mice colonized by the RG2 and the Lupus S107-48 RG strains (**Figure 4C**). There was a numerical trend toward greater intestinal permeability in the female progeny, compared to the male progeny, colonized by the RG2 strain. Compared to controls, a numerical trend towards higher mean levels was also documented in the male mice colonized by the Lupus S107-48 RG strain (*p*=0.057). Strikingly, the highest significantly raised mean levels of intestinal permeability were demonstrated in the female mice colonized with the Lupus S107-48 RG strain (*p*=0.029) (**Figure 4D**). These data demonstrate there is a female bias for induction of impaired gut barrier function resulting from colonization by the S107-48 RG Lupus strain.

### RG translocation to MLN in RG colonized mice with altered intestinal permeability

**Figure 5:**
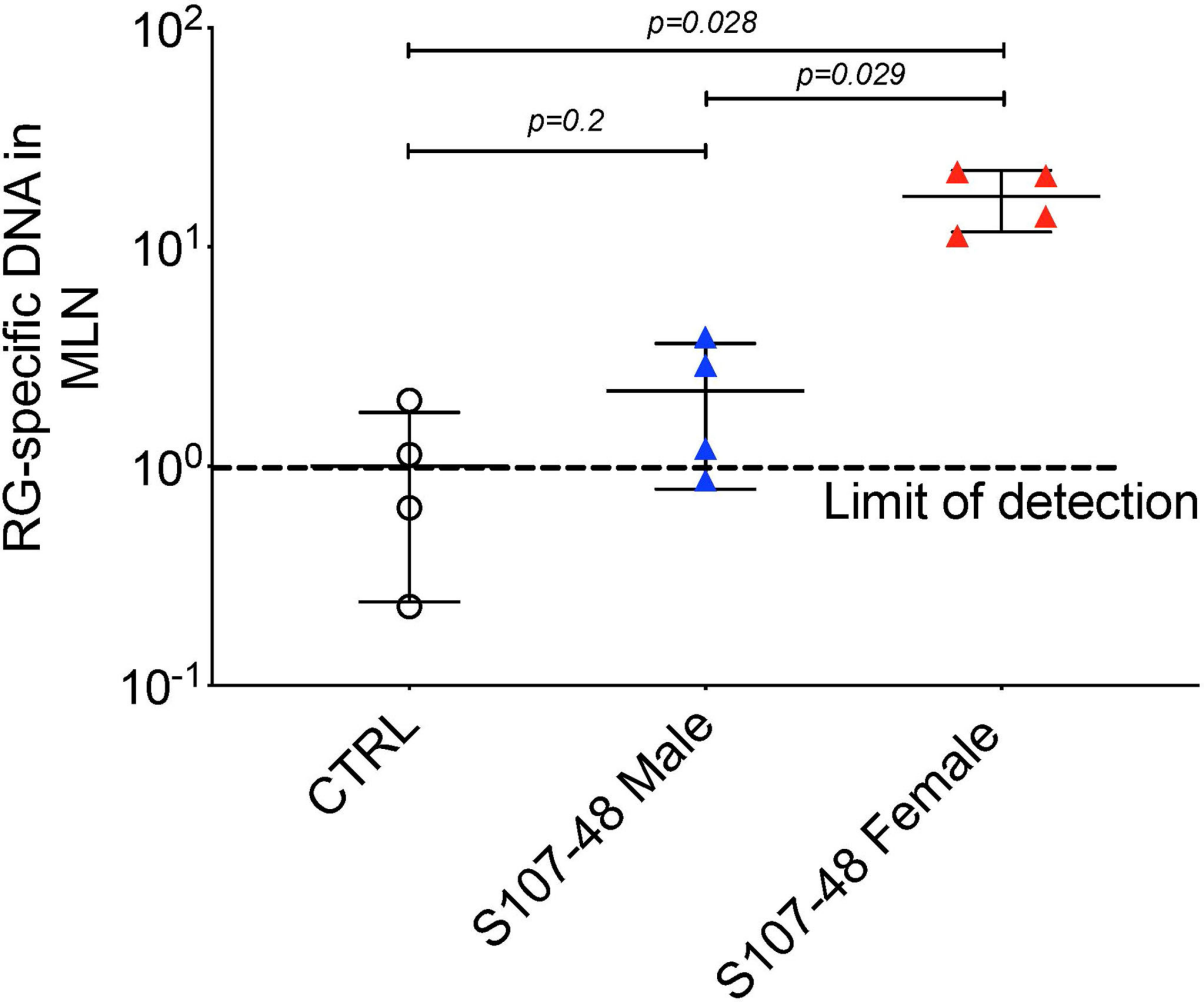
Translocation of S107-48 RG strain 16S rRNA gene DNA in female mice colonized by the S107-48 RG Lupus strain. Extracts of draining mesenteric lymph nodes (MLN) from female 14 wk-old littermates from GF mice colonized with the S107-48 RG strain demonstrate translocated RG genomic DNA into mesenteric lymph nodes (*p*=0.001). Levels in females were significantly higher than in males (*p*=0.002). Control RG2 and S47-18 were also tested at this late time point, but did not attain significance. Comparisons by unpaired *t* test. Methods are described in section *Quantitative PCR analysis*.

Based on above-described evidence of impaired gut barrier, we examined tissue extracts of colonized mice using the RG-specific 16S assay. Reiterating the patterns seen in the intestinal permeability assays (**Figure 4**), after S107-48 strain colonization, we found significant translocation of RG-specific genomic DNA into the mesenteric lymph node in the four female mice (*p*=0.028), with RG DNA levels also significantly above the levels found in four male mice littermates colonized with the same S107-48 Lupus strain (*p*=0.029) (**Figure 5**). Yet, viable bacteria were not recovered. However, persistent RG DNA was not found in splenic extracts, nor was RG-specific 16S DNA detected in mice colonized with RG1 (data not shown). The findings suggest that bacterial components can traverse the gut barrier as a consequence of colonization with some, but not other RG strains, and female mice are much more susceptible to RG-mediated breaches in the gut barrier.

### Systemic immune and autoimmune consequences of RG-mediated breaches of the gut barrier

**Figure 6:**
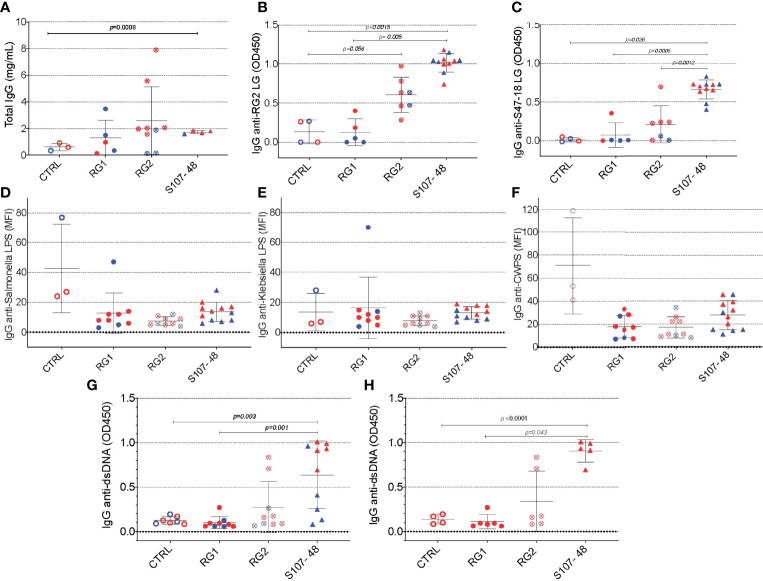
Intestinal colonization with certain RG strains induces IgG RG strain-associated cell wall lipoglycan antibodies and anti-native DNA autoantibodies. **(A)** Following neonatal colonization of litters from RG colonized GF breeding pairs, S107-48 RG strain colonized mice display numerically higher mean serum total IgG levels, compared to controls. **(B)** Neonatal colonization with the RG2 strain or with the S107-48 RG strain induced significantly raised serum levels of IgG anti-RG2 strain cell wall lipoglycan antibodies. **(C)** Neonatal RG colonization does not induce raised IgG-antibody levels to; **(D)** Salmonella LPS, **(E)** Klebsiella LPS, or **(F)** to pneumococcal cell wall polysaccharide (CWPS). **(G)** Neonatal colonization with the S107-48 RG strain induces raised serum levels of IgG anti-native DNA autoantibodies. **(H)** Elevation of IgG anti native DNA following neonatal S107-48 RG strain was greatest in the female mice. (n= 4 to 11 per group). Antibody assays for IgG anti-native DNA used plasma diluted at 1:100 in ELISA (OD450). Antibody assays for IgG antibodies to RG lipoglycans, LPS and CWPS were performed by bead-based multiplex array (MFI) with plasma diluted 1:1000. Comparisons by unpaired *t* test, *p* values shown.

Based on evidence of a breach in the gut barrier in these mice, we evaluated the mice from litters of colonized, previously germ-free mice, for specific systemic immunorecognition of RG-associated antigens. In these studies, we found that RG colonization appeared to raise total IgG levels compared to noncolonized controls (**Figure 6A**), as well as significantly raise levels of serum IgG antibodies in S107-48 colonized mice that recognize the purified RG2 strain lipoglycan as well as the S47-18 strain lipoglycan (**Figures 6B**, **C**). In part, these findings are consistent with antigenic cross-reactivity between lipoglycans isolated from the RG2 and Lupus-associated RG strains (manuscript in preparation), while extracts of the RG1 strain were non-cross-reactive (1). Furthermore, there was no detectable reactivity with LPS isolated from Klebsiella or Salmonella (**Figures 6D**, **E**), nor with pneumococcal cell wall C-polysaccharide (**Figure 6F**), which suggests that the anti-RG reactivity was antigen-specific.

**Figure 7:**
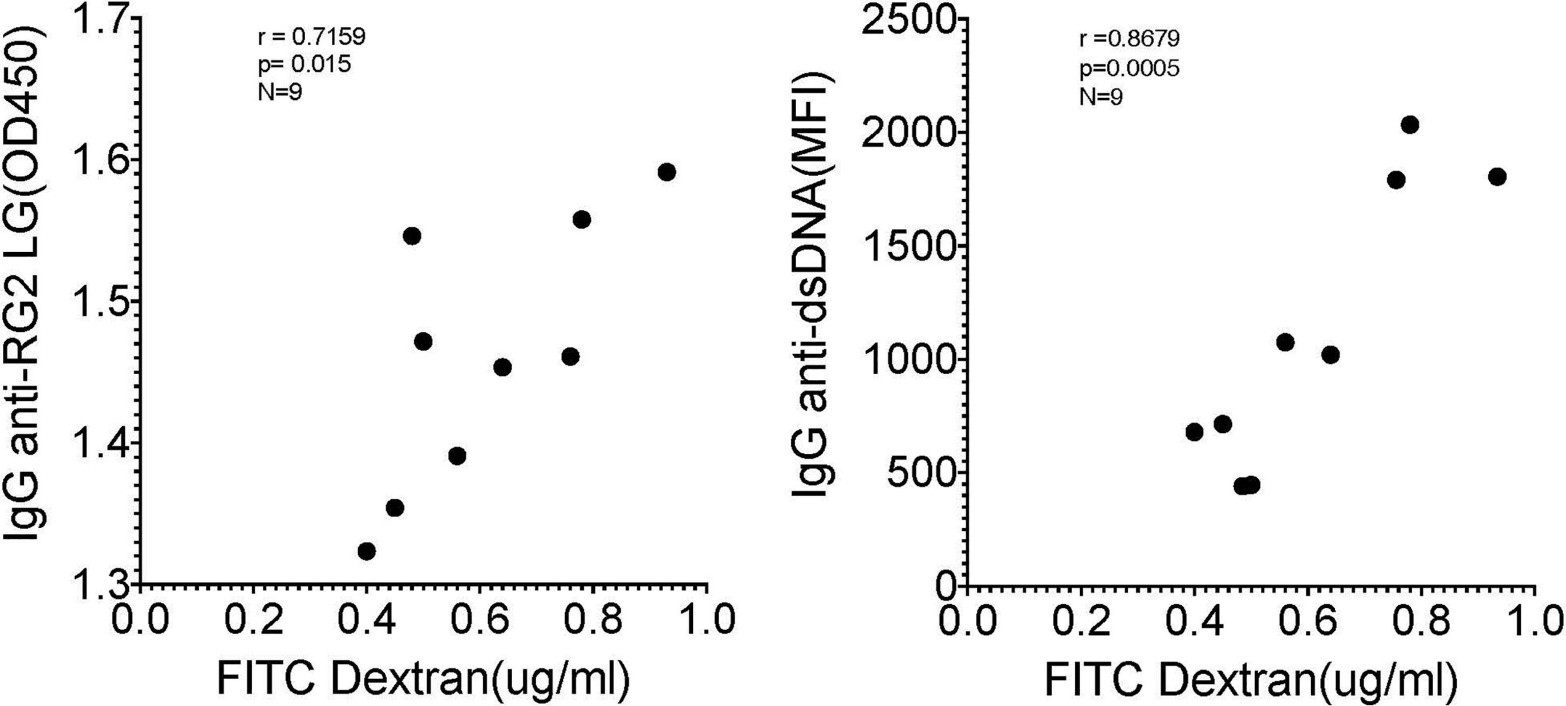
Correlation of levels of IgG anti-RG2 strain cell wall lipoglycan antibodies and IgG anti-native DNA autoantibodies correlate with levels of RG colonization induced increased intestinal permeability. Each point represents an individual male and female littermate that had been neonatally colonized with S107-48 RG strain from a GF breeding pair, as depicted in **Figures 5**, **6B**, **G**, respectively. Results are shown from Spearman correlation analysis. IgG anti-RG2 plasma tested at 1:1000 dilution, and IgG anti-dsDNA was tested at a 1:100 dilution. Samples were studied that were above the 0.22 μg/ml FITC-Dextran (μg/ml) cut-off that was chosen to represent leakiness (i.e., above values in controls)(N=9). Spearman test used, with *r* and *p* values shown.

As there is a reported associations in Lupus patients between levels of circulating IgG anti-RG lipoglycan and Lupus anti-nuclear antibody production (ANA) (1), we assayed plasma samples for binding reactivity with native thymic genomic DNA (dsDNA). These studies demonstrated significantly raised levels of IgG anti-DNA antibodies in mice after neonatal colonization with S107-48 Lupus strain (*p*=0.003) (**Figure 6G**), which was primarily due to the raised anti-DNA levels in the female mice (*p*<0.0001) (**Figure 6H**). Furthermore, in individual S107-48 colonized mice the level of intestinal permeability directly correlated with IgG anti-RG2 lipoglycan antibodies (Spearman r=0.7159, *p*=0.015) and with the level of IgG anti-native DNA autoantibodies (r= 0.8679, *p*=0.0005) (**Figure 7**). These studies therefore document that female mice are more susceptible to Lupus RG induced impairment in the gut barrier, with immunologic consequences of induction of serum antibodies to RG strain-associated lipoglycan determinants, and for Lupus anti-DNA autoantibody production.

**Figure 8:**
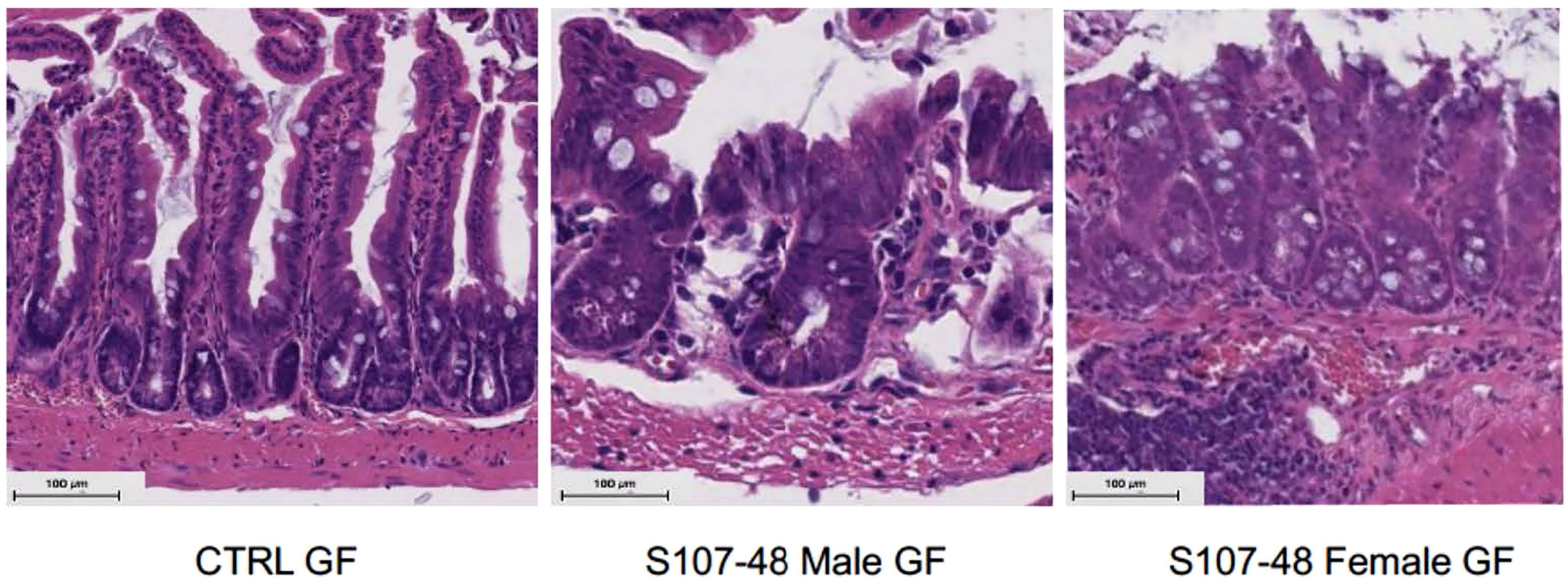
Neonatal RG colonization causes morphologic intestinal abnormalities and raised tight junction Occludin gene transcript levels. Representative H&E staining of terminal Ileum sections from litters of RG-colonized and control or GF mice, with comparisons of mice sacrificed at 14 weeks of age (n = 4 mice/group). S107-48 RG strain colonized mice, which were previously GF, demonstrate subjective flattening of epithelial villi and reduced goblet cells at bases.

To further investigate the mechanisms responsible for increased gut permeability, at 14 weeks of age mice were sacrificed and sections of the small intestine were harvested and histopathologic examinations were performed. These studies demonstrated that unlike the expected morphology of intestinal villi and crypts seen in the control mice raised under GF conditions, that following neonatal colonization with the Lupus S107-48 RG strain the littermates of both sexes demonstrated histologic abnormalities with areas of shortened epithelial villi and changes in the submucosa (**Figure 8**). These findings are consistent with above-described evidence that colonization with some RG strains (i.e., that are lipoglycan producing) induce functional intestinal barrier impairment.

### RG strain-specific increases in gut permeability is reversible with oral treatment with larazotide

The intestinal epithelium is the principal barrier to preserve intestinal compartmentalization and safeguard the host from invasion by enteric bacteria. The physical barrier consists of epithelial and mucus components. The intestinal epithelial layer’s integrity is stabilized by occlusive intercellular molecular joints termed “tight junctions” (TJs) (24). Commensal microbes reinforce the gut barrier through various mechanisms, although increased permeability has been postulated to facilitate entry of bacteria, including their inflammatory product, such as LPS, bacterial DNA and other factors, to cause clinical worsening in predisposed individuals, now implicated in an increasing number of inflammatory and autoimmune disease (25).

Larazotide acetate (formerly AT-001) is a highly polar octapeptide, derived from a prokaryotic *zonula occludens* protein secreted by *Vibrio cholera* (26), has been shown to be an inhibitor of paracellular permeability, and it appears to bind to a receptor on the apical surface of the enterocyte. *In vitro* studies have shown larazotide can prevent the opening of tight junctions, induced by cytokines, bacterial agents and gluten fragments (27, 28).

**Figure 9:**
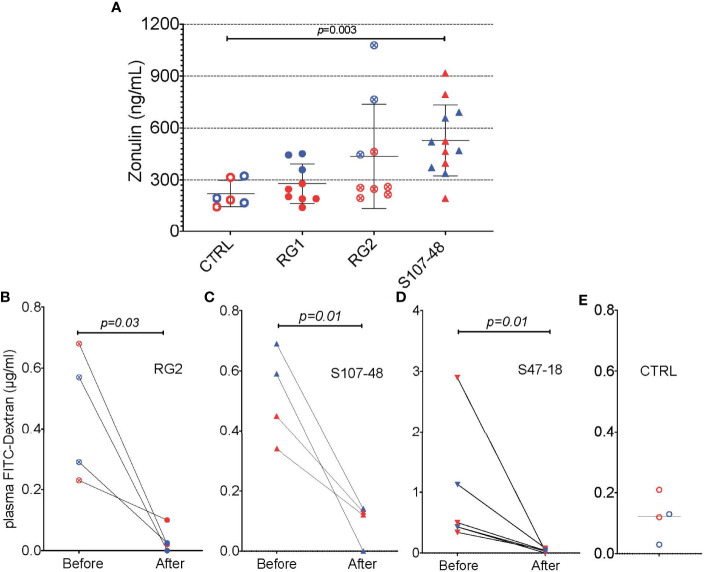
Oral larazotide treatment normalizes increased gut permeability induced by RG strain neonatal colonization. **(A)** Plasma zonulin levels in 14-week-old mice after neonatal colonization, were significantly elevated in S107-48 RG strain mice compared to non-colonized controls (n = 5 to 12 mice). For each group mean+/-SD. In littermates from RG colonized SPF breeding pairs, individual mice were retested with results shown (before), then after a ten-day treatment with larazotide peptide in the water supply and 48 hr rest, gut permeability was then retested (after), with plasma FD4 levels shown for; **(B)** RG2 strain colonized mice, **(C)** S107-48 RG Lupus strain colonized mice, **(D)** S47-18 RG Lupus strain colonized mice, with comparison to **(E)** levels detected in non-colonized control mice. For the bottom panels, significance was tested by paired *t* test. p<0.05 was considered statistically significant.

We therefore wondered whether factors produced by these RG strains, and possibly, but not necessarily, by the RG lipoglycan that has TLR2 agonist activity (1), might affect gut barrier function. We documented that, while RG1 colonization had no effect on serum zonulin levels, there were numerical increases with RG2, and statistically significant increases after Lupus S107-48 strain colonization (p=0.003, **Figures 9A**). We therefore asked whether treatment with larazotide could reverse the increased gut permeability, documented in the FITC-Dex assay, induced by colonization by a lipoglycan-producing RG strain.

To next consider a potential causal role of the elevated serum zonulin levels in the functional abnormalities documented in S107-48 RG strain colonized mice, we repeated the FD4 challenge assay, then treated groups of mice for ten days by oral administration of larazotide acetate (AT-1001). Strikingly, larazotide treatment resulted in normalized gut barrier function, which became completely impervious to the passage of the fluorochrome-labeled dextran compound. Barrier function was normalized in both male and female mice, whether induced by the RG2 strain (*p*=0.03) or the Lupus S107-48 or Lupus S47-18 strains (*p*=0.01 for each) (**Figures 9B**–**D**), compared to control mice (**Figure 9E**). As larazotide is known to act locally to decrease tight junction (TJ) permeability by blocking zonulin receptors to promote TJ assembly and actin filament rearrangement (29), these findings document that gut colonization with Lupus RG strains, result in intestinal permeability, with associated immune responses to microbial and nuclear antigens, which can specifically be inhibited by an agent associated with a molecular pathway known to contribute to celiac disease and to IBD pathogenesis (30).

## Discussion

Whereas recent studies have documented that there are intestinal RG blooms in a subset of patients with active Lupus nephritis (1, 2), the current studies investigated the potential *in vivo* pathogenic properties of RG. As RG is common throughout healthy populations, albeit at lower levels, we reasoned that adverse local effects of intestinal RG colonization on the host may reflect quantitative expansions within intestinal communities and/or arise from genomic variations of these different RG strains. Importantly, diverse RG strains were able to colonize the murine GI tract, including after neonatal RG colonization that must have been mediated by the coprophagic behaviors of the litters from colonized breeding pairs. In part, because adult mice raised under GF conditions are known to have associated developmental immunodefects [reviewed in (31)] this finding was especially important as it enabled the study of mice that were neonatally colonized a natural inter generational fecal-oral passage.

Studies of the potential contributions of gut microbiota imbalances (i.e., dysbiosis) to clinical lupus pathogenesis have been problematic for a number of reasons. Patients in cohorts in microbiome studies reported to date have been highly heterogeneous for clinical features and duration of disease. Hevia et al. (32) reported on a group of 20 patients with SLE, selected for little or no disease activity and off medications, also had only limited imbalances in the ratio of phyla. The issues associated with mixed-sex studies, and recent antibiotic usage, which both affect the microbiota composition, have also been continuing concerns.

An overarching question has been whether dysbiosis, and expansions and/or reductions of certain bacterial species, are truly causative, or whether the local intestinal milieu in a patient with SLE, especially in flare, differentially fosters the outgrowth and competing out of other members of the community. Surveys with 16S rRNA amplimer libraries, or other metagenomic approaches, cannot directly address whether the documented disease associated dysbiosis has a cause or effect role with the clinical condition (33). However, reports have shown that increased systemic inflammation and autoantibody production can be induced by transfer of fecal samples from a disease active Lupus mouse into a germ-free C57BL/6 mouse otherwise not prone to disease (34, 35).

As earlier discussed, the large study of 117 untreated SLE patients has provided clarity that gut dysbiosis is common, and *R. gnavus* blooms are prevalent in LN patients, although not necessarily a consequence of immunomodulatory or antimetabolite agent exposure (2). Murine models have suggested that immune sensitization to lupus-associated autoantigens may be initially primed by immune exposure to autoantigen orthologues in common gut commensals (36). Murine models have also provided evidence that gut barrier defects and translocation of commensals, their antigens, and in some cases live bacteria have been recoverable from draining lymph nodes and the liver (37). However, a subsequent report found sera from both patients with SLE as well as healthy controls had IgG and IgA antibodies reactive with *E. gallinarum* (38), suggesting this commensal is not a common driver of uncomplicated SLE. Taken together, dysfunctional intestinal barrier integrity has been documented in three lupus mouse models (37, 39, 40), while severe lupus-like disease arises in the B6.Sle1.Sle2.Sle3 mouse without evidence of altered gut barrier permeability or commensal translocation (34). Hence, unresolved questions regarding the influence of defined human commensals in SLE patients, especially RG, therefore warranted further study.

We therefore thought it was timely for an experimental design that enabled comparisons of the influences of RG strains isolated from healthy donors, with strain(s) isolated from Lupus patients. To facilitate quantification of the level of RG colonization, we applied a previously reported RG species-specific genomic qPCR assay. This method was adopted due to concern that different strains may differ in the efficiency of *in vitro* culture, which could affect quantitation of recovered colony forming units (CFU), albeit we adopted a 16S rRNA-specific method that cannot discriminate between viable and no longer viable bacterial cells. In any case, our methodologic approach allowed us to address an enigma highlighted by studies of murine colonization by *Enterococcus gallinarum*, which found small intestine colonization in the absence of detectable *E. gallinarum* in the fecal pellets (37). In our studies, at late timepoints after colonization some individual mice displayed persistent substantial level of RG colonization, based on RG detection in cecal luminal contents, but at times had no detectable levels of RG in matched fecal samples. These findings provide a nuanced technical insight into the limits of 16S rRNA gene amplicon analysis of fecal samples which has become widely accepted as an unbiased approach to gut microbiota community analysis.

Our studies demonstrated that colonization with some RG strains, which included CC55_001C (RG2) as well as the S107-48 and the S47-18 Lupus strains reproducibly induced enhanced gut permeability. By contrast, no permeability was induced by RG1 that does not express a lipoglycan. The benign influence of the RG1 strain, from a healthy donor, may be linked to the reported anti-inflammatory properties of the capsular polysaccharide that this strain produces (10). As emerging evidence suggest that subclinical disturbances in gut-barrier function are common in lupus patients (1, 41), the current findings suggest that lipoglycan-expressing RG strains may be direct causes of increased intestinal permeability that contributes to a feed-forward inflammatory autoimmune condition in genetically-predisposed individuals, which contributes to the loss of immune tolerance.

These findings may be especially relevant to understanding the implications of RG blooms during Lupus pathogenesis, a condition that affects nine-fold more women than men (42). Notably, recent reports have shown that female Lupus patients have subclinical abnormalities attributed to breaches in the gut barrier (1) as well as their first-degree female relatives (41). Furthermore, the TJ protein zonulin occludens 1 (ZO-1) has been shown to be expressed at lower levels in females in general, which could suggest that estrogen plays a role on tight junction expression and permeability (43) and discussed in (44). We therefore wonder whether the same mechanisms are responsible in patients as we found in our murine colonization studies. We did not specifically test whether the RG lipoglycan, which was previously shown to contain pathogen-associated molecular patterns (PAMP pro-inflammatory TLR2-agonistic properties (1), directly induced increased intestinal permeability, or whether there could be other RG factors that are (co-)responsible. Admittedly we also did not directly assess whether RG translocation, documented as deposition of RG DNA in the mesenteric lymph node and by the *in vivo* induction of system anti-LG antibodies, was due to leakiness of the small intestine or leakiness of another portion of the gut.

In summary, although there was no consistent pattern of sex-biased intestinal colonization, female mice consistently demonstrated higher levels of intestinal permeability, whether as a consequence of colonization of GF mice, neonatal colonization or after RG colonization of antibiotic-conditioned mice raised under SPF conditions. Indeed, women appear more at risk of intestinal leak (41).

Amongst the most significant findings, impairments of the intestinal barrier function correlated with raised serum levels of zonulin, the only known regulator of intestinal intracellular tight junctions (28). While first discovered in studies of gluten enteropathy, intestinal bacteria (including both pathogens and certain commensals) have also been identified as stimuli that can trigger the release of zonulin (45). Strikingly, the functional intestinal abnormalities induced by lipoglycan-producing RG strains correlated with raised serum zonulin levels, and autoantibody production. Most importantly, the abnormal results of the FITC-dextran assays were completely normalized by oral administration of the specific zonulin receptor antagonist, larazotide that was originally identified from studies of the zonula occludens toxin (ZOT) secreted by *Vibrio cholera* (29). These studies may therefore provide the first mechanistic proof-of-principle for treating Lupus patients with an agent that heals the leaky gut.

## Data availability statement

The original contributions presented in the study are included in the article/**Supplementary Material**. Further inquiries can be directed to the corresponding author.

## Ethics statement

The animal study was reviewed and approved by NYU IACUC.

## Author contributions

GS designed the studies and wrote the first draft of the paper. JD performed the studies and contributed to the writing and editing of the manuscript. DA assisted in the studies, reviewed and edited the manuscript. All authors contributed to the article and approved the submitted version.

## Funding

This work was supported in part by National Institutes of Health Grants P50 AR070591 (GS), R01 A1143313 (mPIs; L. Morel and GS) the Lupus Research Alliance (GS), and the Judith and Stuart Colton Autoimmunity Center (GS).

## Acknowledgments

We appreciate the assistance of the NYU Histology Core Immune Monitoring Core supported by NYU-HHC CTSI Grant UL1 TR000038, and the NYU the Laura and Isaac Perlmutter Cancer Center support grant. We thank Jeffrey Weiser and Martin Kriegel for advice, and acknowledge the staff of Envigo Biologics Inc. for production of the monoclonal hybridoma and antibody.

## Conflict of interest

The authors declare that the research was conducted in the absence of any commercial or financial relationships that could be construed as a potential conflict of interest.

## Publisher’s note

All claims expressed in this article are solely those of the authors and do not necessarily represent those of their affiliated organizations, or those of the publisher, the editors and the reviewers. Any product that may be evaluated in this article, or claim that may be made by its manufacturer, is not guaranteed or endorsed by the publisher.

## Supplementary material

Supplementary Figure 1 In colonization studies into Germ-Free mice, sham gavage or gavage with viable RG1, RG2 and S107-48 was used. In colonization studies with antibiotic pre-treated mice, sham gavage or gavage with Control sham tested viable RG2, S107-48 and S47-18 was used.
